# The Dynamic Arctic Snow Pack: An Unexplored Environment for Microbial Diversity and Activity

**DOI:** 10.3390/biology2010317

**Published:** 2013-02-05

**Authors:** Catherine Larose, Aurélien Dommergue, Timothy M. Vogel

**Affiliations:** 1 Environmental Microbial Genomics, CNRS, Ecole Centrale de Lyon, Université de Lyon, 36 avenue Guy de Collongue, 69134 Ecully, France; E-Mail: timothy.vogel@ec-lyon.fr; 2 Université Joseph Fourier – Grenoble 1 / CNRS, LGGE, 54 rue Molière BP56, F-38402 Saint Martin d’Hères, France; E-Mail: aurelien.dommergue@ujf-grenoble.fr

**Keywords:** Arctic, microbial ecology, biogeochemical cycling, snow

## Abstract

The Arctic environment is undergoing changes due to climate shifts, receiving contaminants from distant sources and experiencing increased human activity. Climate change may alter microbial functioning by increasing growth rates and substrate use due to increased temperature. This may lead to changes of process rates and shifts in the structure of microbial communities. Biodiversity may increase as the Arctic warms and population shifts occur as psychrophilic/psychrotolerant species disappear in favor of more mesophylic ones. In order to predict how ecological processes will evolve as a function of global change, it is essential to identify which populations participate in each process, how they vary physiologically, and how the relative abundance, activity and community structure will change under altered environmental conditions. This review covers aspects of the importance and implication of snowpack in microbial ecology emphasizing the diversity and activity of these critical members of cold zone ecosystems.

## 1. Introduction

### 1.1. The Arctic, a Frozen Ecosystem

A large portion of the Earth is cold: about 14% of the biosphere is polar and 90% (by volume) is cold ocean (less than 5 °C). About two thirds of global freshwater is contained in ice and roughly 20% of the soil ecosystem exists as permafrost [1]. The Arctic, a vast circumpolar area consisting mainly of seasonally ice-covered ocean surrounded by continental land masses and islands, is an important part of the cryosphere, which can be defined as the portion of the Earth where water is in solid form [2]. The Arctic lies above 60°N and is characterized by a harsh climate, unique ecosystems and highly resilient biota [3]. Four million human residents of which approximately 10% are indigenous peoples inhabit many communities in eight countries: Canada, the Kingdom of Denmark (including Greenland and the Faroe Islands), Finland, Iceland, Norway, Russia, Sweden, and the United States of America (Alaska) [3]. 

Seasonal snow cover extends over a third of the Earth’s land surface, covering up to 47 million km^2^ [4] and is also an important feature of the Arctic. Snow cover can be considered as a dynamic habitat of limited duration [5] that acts as a medium and a mediator by transmitting and modifying interactions among microorganisms, plants, animals, nutrients, the atmosphere and soil [6]. Snow cover influences global energy and moisture budgets, thereby influencing climate [4]. The influence of seasonal snow cover on soil temperature, soil freeze-thaw processes, and permafrost has considerable impact on carbon exchange between the atmosphere and the ground and on the hydrological cycle in cold regions [7]. Snow cover acts as both an energy bank by storing and releasing energy and a radiation shield due to its high radiative properties that reflect as much as 80%–90% of the incoming radiation for fresh snow [4]. This high surface albedo reduces absorbed solar energy and lowers snow surface temperature [7].

Snow, a porous media with elevated air content [6], also has a high latent heat of fusion and acts as a heat sink as well as a ground insulator, since heat transfer is poor [4]. The extent and thickness of snow cover influences subsurface soil temperatures and soil metabolic activity [8] and its insulating properties protect soil surface organisms, such as vegetation, invertebrates and mammals against frost damage [4]. Furthermore, snow acts as a reservoir and as a transport medium for liquid water, moves as a particulate flux, and can be relocated by wind [6]. Physical metamorphism, phase changes and chemical transformations, which are modulated by interactions with the atmosphere and soil systems, control both the dynamics and the duration of the snow cover [9]. Thus snow cover is an important factor in the functioning of Arctic, and by extension, global ecosystems.

### 1.2. Snow Formation

Snow is formed in the atmosphere and consists of particles of ice that form in clouds. These crystals grow by vapor deposition and require atmospheric temperatures below 0 °C and the presence of supercooled water [10]. Ice formation is not spontaneous at temperatures above negative 40 °C (233 °K), so ice nucleation occurs mainly in the presence of substrates that act as catalysts. These substrates include dust, seasalt particles, sulfate, combustion products from industrial plants, volcanoes, forests and bacteria [6,11]. A recent report by Christner *et al.* [12] found that biological particles such as proteins or proteinaceous compounds play a significant role in the initiation of ice formation, especially when cloud temperatures are relatively warm. Once deposited, the snow cover forms as a result of snow crystal binding [13]. Snow crystals are subject to temperature gradients that generate water vapor fluxes between crystals. This results in the sublimation of parts of crystals and condensation on other parts, thus changing crystal size and shape and altering the physical properties of the snowpack. With each snowfall, the cover changes and the new layer may possess different properties than the preceding layer [14]. As snow ages, its physical properties, such as density, porosity, heat conductivity, hardness, specific surface area and albedo, evolve in response to thermodynamic stress and weather conditions [13]. Therefore, the composition of layered snow cover and ongoing changes in each of the layers are not only due to the circumstances of formation, but also to changing conditions over time.

### 1.3. Deposition and Incorporation of Impurities within Snowpacks

The snowpack is a receptor surface and storage compartment for nutrients, soluble inorganic, organic matter and contaminants that may or may not be attached to insoluble particles that are delivered by wet and dry deposition (reviewed by [11,15]). Their distribution within the snow is heterogeneous [16] and depends upon different physical processes such as atmospheric loading, wind speed, and snow metamorphism [11]. Nutrients exist in the atmosphere as trace gases such as SO_2_, CO_2_, NO_X_, N_2_O or HNO_3_ and as aerosols such as pollen, sea salt particles, mineral dust and sulfates [11]. Nutrients and contaminants can be delivered to the snowpack through wet and dry deposition. Wet deposition occurs when atmospheric components are scavenged and incorporated into growing or falling snow/rain as condensation or freezing nuclei by either particle impact, gas dissolution or by the collision of supercooled droplets with snow crystals [11]. Condensation and evaporation can alter the concentrations, resulting in the highly variable chemical composition of individual snow crystals. 

Atmospheric scavenging and condensation largely condition the presence of major ions in the snowpack [11]. Dry deposition occurs when gases and particulates are transferred directly to the snow surface without the intermediate scavenging by precipitation. This pathway is dependent upon the atmospheric concentration of the chemical species, the stability or turbulence of the atmospheric boundary layer, as well as the capacity of the surface to retain the chemical species [11]. Once deposited, these species can be redistributed to the snowpack. Due to the permeability of the snowpack, gaseous diffusion occurs along a concentration gradient. Gases can also diffuse from the soil to the atmosphere [5]. Snow-air exchanges occur when the vapor diffuses through the air-filled pore space to the top of the snowpack and from there through a boundary layer to the atmosphere [15]. The penetration of gases and particles through the snowpack is dependent upon its physical-chemical properties, the geometry of the pore space, vapor pressure gradients and wind pressure [11]. Wind advection can accelerate solute transport within snow pores, even when the resistance to molecular diffusive transport is too great to allow gas exchange [15]. 

### 1.4. Snow Metamorphism and Impurity Cycling

The snowpack evolves chemically over time [17]. Physical processes of snow metamorphism also lead to the redistribution of chemical species. On a crystal, molecules diffuse from convex to concave sites, thus transforming crystals to small round snow grains that evaporate and distill onto larger grains once in close proximity. The grains grow rapidly by diffusion, which is initiated by temperature gradients within the snowpack and facilitated by the quasi-liquid surface layer of snow crystals that gives molecules high mobility. During this process, impurities are excluded from the crystals and concentrate at the grain boundaries and pore spaces of the snow [11]. The layered nature of the snowpack, which is composed of a heterogeneous mixture of grains of various sizes, water saturation levels, densities, and ice layers that reduce the permeability to air and water [14], is also important in the redistribution of solutes. Chemicals can be lost from the snow through degradation, volatilization and runoff with meltwater [15]. Impurities can be transformed within the snowpack and also returned to the atmosphere. Snow also transmits atmospherically derived impurities such as nutrients, microorganisms, particles and contaminants to meltwater-fed systems. Snow is, thus, a mediator favoring exchanges among different environmental compartments. 

### 1.5. Snow Melt and Ecosystem Transfer

Melting can occur at air temperatures below 0 °C when solar radiation is intense enough and penetrates into the snowpack [18]. The top snow layers melt first and meltwater percolates downward towards the base of the snowpack. Initially, meltwater is retained in the capillaries and pore walls where it fills 5%–10% of the pore space before becoming more mobile [19,20]. As melting progresses, the water mobilizes solutes and contaminants from the pore walls. Preferential flow may develop due to the non-homogenous nature of the snow and lead to accelerated percolation and solvent concentration of meltwater in certain areas [11]. If the weather conditions prevent further melt, the highly concentrated meltwater may refreeze as a layer within the snowpack and becomes stationary. If multiple freeze/thaw cycles occur, each cycle will increase the solute concentrations of the meltwater, which becomes highly concentrated as it advances deeper into the snowpack [21]. Usually, meltwater reaches the ground, refreezes and develops into a solid layer. The first flush is highly concentrated, with preferential elution of certain solutes [22]. The most soluble ions are removed first and ionic concentrations taper off as melting proceeds [23,24]. Based on laboratory and field studies, fractionation of solutes into meltwater has been shown to occur in all snowpacks, with variable concentration factors [23,25,26]. Roughly 80% of solutes are removed from the snowpack by the first 30% of meltwater [11,27] and fractionation increases with the age of snow, by repeated melting and freezing and slow meltwater flow [23,28,29]. Soluble ions are removed first ([30,31]), followed by, in some cases, the preferential elution of some ions (e.g. SO_4_^2−^, Ca^2+^, Mg^2+^, K^+^, Na^+^) over others (NO_3_^−^, NH_4_^+^, Cl^−^, F^−^) [32]. Species such as non-polar organic molecules are also found in meltwater, but are less easily mobilized by percolating water due to their weak water solubility [33]. Particulate material can also be removed during percolation, but usually remains in the snow until the final stages of melting [33,34,35]. Rain events during the snowmelt period may lead to increases in solute and contaminant load [15]. 

During snowmelt, snow impurities are released to meltwater-fed catchments, soil and aquatic systems, potentially delivering a pulse of highly concentrated solutes, contaminants and microorganisms. Longer melt periods have been shown to lead to increased evaporation of chemicals to the atmosphere, thus reducing contaminant loading to terrestrial and aquatic ecosystems, while short melt periods deliver greater proportions of stored contaminants [15]. The snow cover lasts several months, thereby leading to longer solute accumulation periods, and arctic ecosystems are especially at risk for pulse exposure since the melt period is short [15]. 

## 2. Biology of the Cryosphere

### 2.1. Colonization and Activity in Cold Environments

Microorganisms exist in several extreme cold environments such as glacial ice [36,37,38], sea ice [39], Arctic biofilms [40], Arctic snow [41,42], supercooled clouds [43] and Antarctic permafrost [44]. Although both poles are different, it is likely that some of the microbial colonization pathways described for Antarctica, such as atmospheric circulation, ocean currents, birds, fishes, marine mammals and human vectors apply to the Arctic as well [45]. Aerial transport has long been viewed as a major transport route given that spore formers, such as Gram-positive bacteria and fungi, are able to survive long-range transport [46]. Due to the cold conditions and the limited supply of liquid water, snow and ice have long been only considered as entrapment and storage systems for microorganisms that were thought to enter as vegetative and resting cells, transported by wind-blown particles, aerosols and ice crystals. These cells would then be buried by subsequent snowfall events before being transferred to other systems upon snowmelt [47]. However, this view started to change with a number of studies that examined microbial diversity, ecology and function in the cryosphere. Whether the microorganisms found in cold environments are metabolically active and reproducing remains unclear, but it is assumed that certain microbial species are at least able to survive [2]. 

The occurrence of related phylotypes from geographically-diverse cold environments has been reported [39], suggesting that adaptation for survival, persistence and activity at low temperatures might be a common feature of these species and that they might possess common adaptive strategies [1]. The bacterial classes most frequently reported are *Proteobacteria* (*Alphaproteobacteria*, *Betaproteobacteria* and *Gammaproteobacteria*), *Bacteroidetes* group, low and high G+C Gram-positive genera, and *Cyanobacteria* [1,37,48,49]. Moreover, microorganisms might be metabolically active at low temperatures down to −20 °C [50,51] and very low rates of metabolic activity might be sustained for up to 10^4^ to 10^6^ years and at temperatures as low as −40 °C [52]. 

However, these studies focused on characterizing bacteria in ice or permafrost and relatively little is known about life in snow, despite the extent and importance of seasonal snow. The snow cover might support a microbial community composed of snow algae, bacteria, yeasts and snow fungi [5,53,54]. Snow algae represent an ecologically and physiologically specialized group that can form visible blooms, however their development is dependent on the availability of liquid water and are only active during the spring and summer, when air temperatures are above zero (reviewed in [54]). Snow algae have been studied relatively extensively [54,55,56,57], but data on bacteria inhabiting seasonal snow cover are sparse, especially for polar snowpacks. Carpenter (2000) reported low rates of DNA synthesis and the presence of Thermus-Deinococcus-like organisms in Antarctic snow [58], while Amato *et al.* (2007) used culture-based methods to isolate 10 bacterial strains belonging to *Proteobacteria*, *Firmicutes* and *Actinobacteria* from a snowpit dug on a polythermal glacier in Svalbard (Norway) [41]. Both studies focused on bacterial density and activity, but important questions about diversity, community structure, population dynamics and function remain unanswered. Using a 16S rRNA gene (rrs) clone library approach on snow and meltwater from Svalbard, Larose *et al.* (2010) observed high levels of diversity, similar to those of Arctic pack ice and Arctic microbial mats [39,42]. Significant differences in diversity among sample types were also reported and these may be related to seasonal changes in the snow environment (*i.e.*, pH, water content and temperature). Segawa *et al.*, 2005, also observed seasonal changes in bacterial flora and biomass in mountain snow in Japan, with increases in biomass during the melting season (March to October) that were attributed to nutrient and/or environmental conditions in the snow [59]. These results highlight the links between environmental conditions and changes in community structure.

The snowpack appears to be a diverse habitat and many studies suggest the occurrence of related phylotypes from geographically diverse, but predominantly cold environments [42,46,59]. However, the seasonal evolution of the microbial community and the physiological state of the organisms within the snowpack are topics that remain to be addressed. 

### 2.2. Life in the Cold Lane

In order to colonize and survive in cold environments such as snowpacks, microorganisms must overcome a number of physiological stress parameters such as cold temperatures (less than 5 °C), high levels of solar radiation, desiccation and freeze/thaw cycles [1]. These harsh environmental conditions vary temporally as well as spatially and necessitate physiological acclimation. In the Arctic, because of the high latitudes, a pronounced seasonality causes gradual, yet extreme, changes in the photoperiod, irradiance, and temperature. During the springtime melt period, snow undergoes temperature shifts across the freezing point of water, leading to a more dynamic environment, but also to an increase in freeze/thaw cycles [60].

Different survival strategies at low temperatures have been observed in bacteria: reduction of cell size and capsular polysaccharide coat thickness, changes in fatty acid and phospholipid membrane composition, decrease of the fractional volume of cellular water, increase of the fraction of ordered cellular water, energy synthesis by catalysis of redox reactions of ions in aqueous veins in ice or in thin aqueous films in permafrost [52]. Moreover, many species that have been isolated form spores that provide high resistance levels, while others have thick cell walls or polysaccharide capsules that resist freeze/thaw cycles [1]. Cold tolerance has been shown to involve down-regulation of enzymes involved in major metabolic processes such as glycolysis, anaerobic respiration, ATP synthesis, fermentation, electron transport, sugar metabolism as well as the metabolism of lipids, amino acids, nucleotides and nucleic acids [61]. However, up-regulation and overexpression of several enzymes and proteins (cold shock proteins, *etc.*) may enhance survivability during freeze-thaw cycles [61]. Other adaptive strategies include the production of pigments such as oligosaccharide mycosporine-like amino acids, scytonemins, carotenoids, phycobiliproteins and chlorophylls that offer a broad strategy to cope with high irradiance [60].

Ability to attach to surfaces also provides bacteria with adaptive strategies. Junge *et al.* (2004) reported that particle-associated bacteria were more active than free-living cells as temperatures dropped and that they also produced exopolysaccharides (EPS). Bacteria growing in microbial mats were also shown to form EPS [60]. The EPS production favors attachment [51] and protects against freezing, dessication, viral and bacterial attacks [60]. Moreover, it is likely that the presence of species with specialized mechanisms of stress resistance may provide a protective effect on other members of the community. For example, certain pigments such as oligosaccharide mycosporine-like amino acids and scytonemin are located outside the cells and may benefit non-producing microorganisms against radiation damage [60]. 

### 2.3. Microorganisms—Active Members of the Cryosphere?

Recent reports suggest that microorganisms impact nutrient dynamics, composition and abundance [16], that they may shift surface albedo of snow and ice [62,63] and that they impact hydrochemistry [64]. Critical processes controlling biogenic trace gas (e.g., CO_2_, CH_4_, N_2_O, and NO) fluxes are carried out by microorganisms [65]. Within the snowpack, microbiological activities such as carbon fixation by algal communities may modify the nutrient cycle [5]. The importance of bacteria in governing redox conditions and their role in Fe, S, N and P cycling is now acknowledged [16]. The role of bacteria in carbon cycling in Antarctic surface snow has recently been highlighted. Antony *et al.* (2012) reported that snow bacteria were able to use a wide range of low and high molecular weight carbon substrates and suggested that these organisms could potentially govern snow chemistry [66]. Microorganisms may also be responsible for the metabolism and transformation of contaminants such as pesticides [67] and mercury [40,68], both pollutants of arctic ecosystems. Mercury is an excellent example of the possible importance of microorganisms in pollutant fate in snowpacks.

The Arctic is experiencing mercury (Hg) toxicity [68] and Hg concentrations are increasing. Mercury exists in several forms in the environment: elemental (Hg°), divalent form (Hg^2+^) and an organo-metallic form of which methylmercury (MeHg) is the most important. The MeHg organic form is the most toxic of the three forms, even at very low exposure doses [69]. Mercury is mainly emitted to the atmosphere in its gaseous form (Hg°), but also in the oxidized form (reactive gaseous mercury, RGM) or in the particle-bound form (particulate mercury, PM). Hg° has a relatively long atmospheric residence time (between 0.5 and 1.5 years) and average atmospheric concentrations have been estimated to 1.7 g/m^3^ for the Northern Hemisphere [70]. RGM and PM have shorter lifetimes and tend to be deposited near their sources [70]. Mercury reaches polar ecosystems mainly as Hg°; however due to the cyclical nature of Hg transformations (transport-deposition-re-emission), even mercury originally emitted as RGM and PM can be transported to the Arctic [71]. Similar to other contaminants, Hg can be deposited after atmospheric scavenging by precipitation and dry deposition. Once RGM is formed in the atmosphere, snow can act as an efficient surface for its sorption. In addition, active growth of snow and ice crystals from the vapor phase readily scavenges available RGM [72]. 

In 1995 at Alert, Canada, Schroeder *et al.* (1998) measured the episodic near-total depletion of Hg° from the atmosphere during the spring [73]. These events, termed Atmospheric Mercury Depletion Events (AMDEs), were observed in parallel to the depletion of ozone [74] and led to intense field, laboratory and theoretical studies to determine which reactions were involved. In particular, mercury was shown to undergo rapid oxidation and deposition via photochemically-initiated reactions believed to involve reactive marine halogens, mainly Br and BrO [75,76,77]. These reactions transform Hg° to PM and RGM species that can then be deposited onto the snow. It has been estimated that AMDEs increase polar mercury deposition by 100 tonnes a year [71], yet the post-depositional fate of this Hg remains uncertain, although it could undergo a series of possible transformations once deposited [78]. Using a bacterial *mer-lux* biosensor, Larose *et al.*, 2011 detected Hg in Arctic snow in a bioavailable chemical form, *i.e.* able to interact with microorganisms, and showed that fresh snowfall events contributed to higher proportions of BioHg than mercury depletion events [79]. Therefore, Hg is deposited in a chemical state that allows for biological uptake and transformation. Different simultaneous biotic and abiotic processes alter the chemical state of mercury and thereby its toxicity in the environment. Four different reactions control mercury speciation: methylation, demethylation, reduction and oxidation [80] and microorganisms can carry out each of these transformations. Microorganisms are able to methylate mercury. Bacteria have been isolated from Arctic snowpacks [41] and microbial activity has been measured at temperatures down to −20 °C [50]. Constant *et al.* 2007, reported increases in the MeHg:THg ratio and positive correlations with bacterial colony counts and particles. These results led to the hypothesis that MeHg was being formed within the snowpack, despite the absence of correlation with sulfate-reducing bacteria (SRB), the principal methylators in anoxic environments [81]. Recently, Larose *et al.*, 2010 proposed a mechanism by which bioavailable Hg may undergo methylation by microorganisms in an aerobic process involving biogenic sulfur molecules [17].

In order to cope with the toxicity of Hg and MeHg, bacteria have developed specialized resistance mechanisms. For example, bacteria possessing the *mer* operon are able to detoxify Hg via MerA [80]. The genes that encode MerA have been isolated both from a variety of environments including soil [82] (150), Siberian permafrost [83] and Arctic biofilms [40] and from bacteria [80] and archaea [84]. Some bacteria are able to detoxify both BioHg and MeHg, while others are only able to transform inorganic mercury via the *mer* operon resistance pathway [80]. Based on results from a cultivation study of Arctic snow bacteria, Moller *et al*, 2011 were able to demonstrate that mercury-resistant bacteria accounted for almost a third of cultivatable organisms and that 25% of these were able to completely reduce mercury, thus limiting the supply of Hg available for methylation [85]. These mercury resistant bacteria may therefore help lower the risk of methylmercury entering Arctic food chains.

## 3. General Conclusion and Perspectives

The Arctic climate is changing. A 20^th^ century warming trend has been documented in the Arctic, with air temperatures over land areas increasing by as much as 5 °C and increased temperatures over sea ice [3]. In addition, precipitation has also increased. Other changes include a 2.9% per decade decrease in Arctic sea-ice extent (1978–1996), the thinning of sea-ice, an increase in melt days par summer, the warming of Atlantic water flowing into the Arctic Ocean, the thinning of the oceanic surface layer and the increase in ground temperatures and resulting permafrost melt [86]. Decreases in snow and ice cover, increased plant growth, increased primary production of terrestrial algae in freshwater lakes, and the northward movement of the tree line in the most-warmed Arctic regions have also been reported [3]. These changes are probably linked to human activities that are clearly influencing the climate, with arctic (and polar) environments subjected to substantial warming and increases in precipitation over the 21^st^ century. 

Climate change is also expected to alter contaminant loading and transformations in the Arctic. The predicted warming of air temperatures at lower latitudes will have direct effects on contaminants through increased volatility, more rapid degradation and altered partitioning between phases [87], while increased precipitation could lead to more scavenging of contaminants by rain and snow, thereby augmenting inputs to aquatic and terrestrial ecosystems [3]. Extended ice-free areas in the Arctic Ocean may favor both atmospheric scavenging by precipitation in addition to seawater partitioning [3] and certain contaminants might evade from surface seawater more rapidly [87].

While it appears that microbial life is well adapted to cold ecosystems, the response of these populations to changing environments is mostly unknown. Climate change may alter microbial functioning by increasing growth rates and substrate use due to increased temperature. This may lead to changes in process rates. Another impact could be the restructuring of microbial communities [65]. Biodiversity may increase as the Arctic warms and population shifts occur as non-heat tolerant species disappear in favour of more heat tolerant ones. For example, a circumpolar shift was seen in the fossil remains of algae and invertebrates in the mid to late 19^th^ century probably due to climate change. In order to predict how ecological processes will evolve as a function of global change, it is essential to identify which populations participate in each process, how they vary physiologically, and how the relative abundance, activity and community structure will change under altered environmental conditions [65]. 
